# Non-Invasive Ultrasound Diagnostic Techniques for Steatotic Liver Disease and Focal Liver Lesions: 2D, Colour Doppler, 3D, Two-Dimensional Shear Wave Elastography (2D-SWE), and Ultrasound-Guided Attenuation Parameter (UGAP)

**DOI:** 10.7759/cureus.72087

**Published:** 2024-10-22

**Authors:** Andreas I Biris, Ioannis Karamatzanis, Despoina Biri, Ioannis A Biris, Nikolaos Maravegias

**Affiliations:** 1 Clinical Teaching Fellow, Southend University Hospital, Mid and South Essex National Health Service (NHS) Foundation Trust, Southend, GBR; 2 Internal Medicine, Basildon University Hospital, Basildon, GBR; 3 Psychiatry, Royal Edinburgh Hospital, National Health Service (NHS), Lothian, GBR; 4 General Surgery, Ultrasonography, Yperichos, Piraeus, GRC; 5 Internal Medicine, Athens Medical Centre, Athens, GRC

**Keywords:** liver ultrasound, metabolic dysfunction-associated steatohepatitis (mash), metabolic dysfunction-associated steatotic liver disease (masld), shear-wave ultrasonography, steatotic liver disease, two-dimensional shear wave elastography (2d-swe), ultrasound-guided attenuation parameter (ugap), ultrasound (u/s)

## Abstract

We conducted a comprehensive literature review to evaluate the efficacy of combining two-dimensional shear wave elastography (2D-SWE) and ultrasound-guided attenuation parameter (UGAP) in assessing the risk of progressive metabolic dysfunction-associated steatohepatitis (MASH). This narrative review explores the applications of liver ultrasound in diagnosing metabolic liver diseases, focusing on recent advancements in diagnostic techniques for steatotic liver disease (SLD). Liver ultrasound can detect a spectrum of SLD manifestations, from metabolic dysfunction-associated liver disease (MASLD) to fibrosis and cirrhosis. It is also possible to identify inflammation, hepatitis, hepatocellular carcinoma (HCC), and various other liver lesions. Innovative ultrasound applications, including elastography and UGAP, can significantly enhance the diagnostic capabilities of ultrasound in accurately interpreting liver diseases. Understanding the pathogenesis of liver diseases requires a thorough analysis of their etiology and progression in order to develop sound diagnostic and therapeutic approaches. Chronic liver diseases (CLD) vary in origin, with MASLD affecting approximately 20-25% of the general population. The insidious progression of CLD from inflammation to fibrosis and cirrhosis underscores the need for effective early detection methods. This review aims to highlight the evolving role of non-invasive ultrasound-based diagnostic tests in the early detection and staging of liver diseases. By synthesizing current evidence, we aim to provide an updated perspective on the utility of advanced ultrasound techniques in redefining the diagnostic landscape for metabolic liver diseases.

## Introduction and background

The diagnosis and management of metabolic liver diseases rely heavily on the expertise of the attending physician. Following a thorough history-taking and clinical examination, it is crucial to investigate the extent and nature of disturbance in the liver parenchyma. This process involves requesting appropriate laboratory tests, including haematological assessments, imaging studies, endoscopic evaluations, and biopsies as needed in order to establish a differential diagnosis of liver disease [1].

Imaging modalities used for the assessment of liver disease include ultrasound, computed tomography (CT), and magnetic resonance imaging (MRI) [2]. Among these, ultrasound is the method of choice based on several advantages: A) Availability: Ultrasound is widely accessible, and increasing numbers of clinicians and radiographers are becoming adept in its use. B) Cost-effectiveness: It is relatively inexpensive compared to other imaging modalities such as CT and MRI. C) Safety: It does not expose the patient to ionising radiation, allowing for repeated use if needed. D) Immediate results: This imaging modality offers real-time imaging and allows for prompt clinical decision-making to be made. E) Versatility: It can be used for the diagnosis of a wide variety of clinical conditions, including liver fibrosis, cystic lesions, or abnormalities of the biliary tree [3].

Elastography is a non-invasive imaging technique that primarily assesses the progression of diffuse liver diseases, including fibrosis due to chronic hepatitis and cirrhosis. It enables the staging of liver disease and the monitoring of treatment responses without the need for more invasive methods such as biopsies [4]. The combination of elastography and Ultrasound-Guided Attenuation Parameter (UGAP) can significantly enhance the quantification of hepatic steatosis, especially in cases of metabolic dysfunction [5].

Steatotic liver disease (SLD) is characterised by excessive fat accumulation in the liver parenchyma, which can either be benign or more serious and lead to liver damage. Formerly known as non-alcoholic fatty liver disease (NAFLD), this condition is now classified as Metabolic Dysfunction-Associated Liver Disease (MASLD). MASLD is defined by hepatic steatosis exceeding 5% of hepatocytes in the absence of significant alcohol consumption or other known liver disease causes and is typically accompanied by at least one metabolic risk factor [6].

MASLD is projected to become the leading cause of liver transplantation, potentially surpassing hepatitis C [7]. When metabolic dysfunction coexists with increased alcohol consumption, the condition is classified as metabolic dysfunction and alcohol-related liver disease (MetALD). This differentiation is crucial, as alcohol alone can lead to steatotic liver disease, resulting in steato-necrosis and liver fibrosis [8]. The primary risk factor for MASLD is metabolic syndrome, which is characterised by the presence of at least three of the following five criteria: Abdominal obesity, hypertension, hyperglycemia, high serum triglycerides, and low HDL cholesterol. MASLD is currently the most prevalent liver disorder globally, affecting approximately 25% of the population, particularly in developed countries [9]. Almost 60% of individuals with diabetes and up to 10% of individuals within the “normal” weight range may go on to develop MASLD [10]. Depending on the severity and progression, the condition can range from simple hepatic steatosis to Metabolic Dysfunction-Associated Steatohepatitis (MASH), which can lead to complications such as fibrosis, cirrhosis, and hepatocellular carcinoma due to hepatocyte apoptosis, inflammation, and fibrosis [11]. According to the literature, it is emphasised that patients with an established diagnosis of MASH face an increased risk of liver-related mortality, underscoring the critical importance of monitoring hepatic steatosis for early diagnosis, treatment, and follow-up of patients with MASLD [12].

Liver fibrosis results from an active response to chronic liver injury lasting more than six months, leading to chronic liver disease (CLD). The histological assessment of the location, extent, and severity of fibrosis is vital for determining the disease stage and serves as a key prognostic indicator for the progression of chronic liver diseases. Ongoing liver damage and subsequent fibrosis progression disrupt the liver's architecture, potentially leading to cirrhosis and liver failure. Fibrogenesis is a dynamic process involving the remodelling of the extracellular matrix (ECM), characterised by collagen breakdown and synthesis. Stellate cells (Ito cells) in the liver play a central role in the development of fibrosis, transforming into myofibroblasts upon activation. This activation can be triggered by various factors, including cytokines and growth factors, following liver injury [13].

Common causes of liver fibrosis include chronic hepatitis B and C infections, metabolic dysfunction-associated liver disease (closely associated with obesity and insulin resistance), chronic metabolic conditions (such as diabetes mellitus), alcohol-related liver disease (ALD), autoimmune liver diseases, toxins, and excessive iron and copper intake [11].

The assessment of liver fibrosis is crucial, as patients with MASLD and advanced fibrosis are at increased risk of progressing to end-stage liver disease. Non-invasive composite scores can help determine whether patients with CLD have significant or advanced fibrosis. These scores can guide clinical management decisions, with higher scores indicating the need for specialist referral. The increasing prevalence of metabolic liver dysfunction and its complications underscores the need for effective diagnostic and monitoring tools. Advances in imaging techniques, particularly ultrasound elastography and UGAP, offer promising avenues for improving the management of liver diseases [14]. This review article aims to evaluate the role of ultrasound elastography and Ultrasound-Guided Attenuation Parameter (UGAP) in the diagnosis, assessment, and monitoring of metabolic dysfunction-associated liver disease (MASLD) and liver fibrosis, highlighting their potential to improve patient management and outcomes.

## Review

A liver biopsy is typically required to confirm the diagnosis of fatty liver disease. Pathological examination of a sufficient liver sample certifies and stages parenchymal fibrosis. Ultrasound-guided liver biopsy, however, has numerous limitations as it provides information from a small sample that may not be representative of the entire liver. Additionally, it is quite an invasive procedure, associated with risks around it, and dependent on the examiner's skill. As a result, the development of non-invasive methods to assess and monitor liver tissue quality and fibrosis progression is crucial. Recent advancements include MRI-based Proton Density Fat Fraction (MRI-PDFF), a non-invasive, quantitative biomarker for assessing liver fat content. However, limited accessibility and high costs restrict its widespread use, especially for routine follow-ups [15].

Hepatic steatosis, characterised by lipid accumulation in the liver, is a common liver condition. Non-invasive diagnostic markers include serum markers and imaging techniques. Ultrasound-based methods offer simpler and safer alternatives for detecting and staging liver disease. While these methods cannot yet differentiate MAFLD from MASH, they are valuable for evaluating patients with steatosis without advanced fibrosis [16]. This review focuses on imaging techniques for detecting and monitoring SLD using ultrasound, particularly elastography and ultrasound-guided attenuation parameter (UGAP), which estimate liver stiffness and elasticity.

Two-dimensional (2D) ultrasound uses high-frequency sound waves (2 to 24 MHz) to create images of internal or superficial organs. It is widely used to examine the texture of solid organs such as the liver, gallbladder, pancreas, and spleen, as well as other abdominal and pelvic organs as well as soft tissues [3].

Colour Doppler scans (Triplex) have enhanced ultrasound diagnostics by allowing visualisation of blood flow in blood vessels within organs. This technology has improved hemodynamic studies of the heart, carotids, abdominal aorta, and peripheral vessels. Power Doppler enables detailed studies of circulation in small vessels, aiding in the assessment of nodules [3]. Modern ultrasound technology also offers microvascular imaging (MVI) for high-definition imaging of small structures [17].

Three-dimensional (3D) ultrasound represents a significant advancement, providing volumetric information that benefits interventional procedures. It complements 2D ultrasound, which is commonly used for diagnosis and guided needle biopsy of suspicious masses. 3D ultrasound acquisition is based on mechanical, electromagnetic, and free tracking of conventional 2D ultrasound transducers or 2D array transducers [18].

Conventional imaging methods cannot be relied upon to provide information about the mechanical behaviour of the examined tissues. Tissue elasticity can be altered by a variety of factors, such as tissue injury, tumour development, or diffuse fibrosis. Recent advancements in ultrasound technology have enabled the non-invasive assessment of soft tissue mechanical properties in vivo through the use of elastography [19]. A key principle in elastography dictates that upon applying a given force to a tissue, softer tissues will deform more compared to harder tissues. Analogous to palpation, elastography techniques take advantage of the effect of liver fibrosis on the tissue, which makes it more inflexible, and quantify tissue response to mechanical stimuli by measuring the shear wave velocity or tissue displacement induced by ultrasonic or physical force, providing qualitative and quantitative measures of said liver tissue stiffness [20].

Vibration-controlled transient elastography (VCTE or Fibroscan) and real-time elastography (RTE) use a mechanical guide to generate a shear wave and measure its velocity using Doppler ultrasound or magnetic resonance (MR) techniques, respectively. MR techniques and ultrasound elastography are combined through the use of magnetic waves with low-frequency liver ultrasound. The combination of these imaging techniques yields a visual map (elastogram), which depicts any point of increased stiffness within the liver tissue [21].

Point shear wave elastography (pSWE, or acoustic radiation force impulse) and two-dimensional shear wave elastography (2D-SWE) employ high-frequency ultrasound to generate shear waves. pSWE measures the shear wave generated by an ultrasound frequency in meters per second, while 2D-SWE measures ultrasound waves at multiple frequencies in real-time in kPa using two-dimensional ultrasonography. Finally, real-time elastography (strain) uses standard ultrasound to measure the displacement (or strain) of liver tissue caused by the ultrasound transducer. It is important to highlight that due to the different natures of the aforementioned techniques, elastography values yielded by different techniques are not comparable, as they are measured in different units [22].

Two-dimensional shear wave elastography (2D-SWE) represents a significant leap in this field as it allows real-time visualisation of tissue stiffness on conventional 2D ultrasound images using a colour-coded overlay. This technique quantifies tissue hardness, offering valuable insights for diagnosis, staging, and management of diseases that alter tissue elasticity [3]. In shear wave elastography, the ultrasound transducer generates an acoustic radiation force impulse (ARFI) that induces a shear wave in the tissue. The propagation speed of this wave, measured at different lateral positions, correlates with tissue stiffness. This principle allows for the quantitative assessment of liver fibrosis and cirrhosis, with results comparable to those obtained from liver biopsy. Elastography techniques can be classified based on the type of information imaged, the source of mechanical stimulation, and the imaging modality used to monitor tissue response. For liver fibrosis assessment, several ultrasound elastography techniques have been developed: transient elastography (VCTE), real-time elastography (RTE), acoustic radiation force impulse (ARFI), and shear wave elastography (SWE) [23]. It is worthwhile to highlight that SWE has been shown to have a lower operator error rate when compared to TE by 3 to 16% [24].

While conventional imaging methods provide structural information, they lack insight into tissue mechanical properties. Elastography fills this gap, offering crucial data on tissue elasticity, which can be altered by various pathological processes, including inflammation, fibrosis, and neoplasia. The applications of elastography extend beyond liver fibrosis evaluation in chronic liver disease. It shows promise in predicting cirrhosis complications and monitoring treatment responses in chronic viral hepatitis. As the technology continues to evolve, elastography is poised to play an increasingly important role in non-invasive differential diagnosis across multiple organ systems [25]. Nowadays, elastography is also being employed to investigate thyroid nodules, mammary glands, and soft tissues, especially tendons [26]. In general, liver elastography is a specialised, non-invasive investigation for the assessment of liver damage, and its degree of reliability lies close to that of liver biopsy, which has long been considered the gold standard since it is painless, easily reproducible, and provides immediate results [27]. In liver fibrosis, liver damage can be staged according to either the degree of histological damage accrued by the tissue or the elastography findings, as shown in Table 1 [28].

**Table 1:** Staging of fibrosis according to histological image and SWE

Histological Findings	Liver Stiffness Result	Fibrosis Score
No visible fibrosis and perisinusoidal/portal/periportal fibrosis	2 to 7 kPa	F0 to F1
Perisinusoidal and portal/periportal fibrosis	7 to 11 kPa	F2
Bridging fibrosis between adjacent portal tracts or between a portal tract and an adjacent hepatic venule [29]	11 to 14 kPa	F3
Cirrhosis, which can lead to cell necrosis and liver failure	14 kPa or higher	F4

Elastography may be used to assess liver disease in collaboration with hepatologists and hepatologic centres. Such a programme was launched in Greece in 2016 by the Hellenic Liver Patients Association and mainly involved assessing patients in rehabilitation facilities and prisons. According to the results of this campaign, 6,623 examinations using Fibroscan liver elastography were carried out between April 2016 and March 2018. The liver diseases involved in those tests were Hepatitis C 3,697 (56%), Hepatitis B: 943 (14%), NAFLD/MASLD: 543 (8%), Primary Biliary Cholangitis (PBC): 139 (2%), Alcoholic Hepatitis (ALD/MetALD): 124 (2%), Autoimmune Hepatitis (AIH): 76 (1%), and other diseases of the liver: 1,101 (17%). More specifically, out of the 3,697 patients with hepatitis C, 49% of them were diagnosed with a liver fibrosis score of F0-f1, 23% with a score of F2, 12% with a score of F3, and 16% with a score of F4. This programme was carried out in 13 regions across the country, did not put extra pressure on the National Health Service, and continues to this day [30].

Another important application of elastography is found in the monitoring of patients with primary biliary cholangitis (PBC) [31]. According to the guidelines set by the European Association for the Study of the Liver in 2017, static liver stiffness measurement (LSM) over 9.6 kPa can distinguish patients with a fivefold risk of developing further complications. According to the latest data, it is now possible to further risk stratify such patients as follows: Patients with LSM values <8 kPa show a small risk of complications; LSM values of 8-15 kPa show moderate risk; and LSM values ≥15 kPa show the greatest risk of developing complications [32]. Recent studies have shown that patients responding to treatment with LSM values <10 kPa and alkaline phosphatase values within the normal range have a 10-year survival >90% [33]. Data remains limited, however, on the prognostic value of LSM changes during monitoring patients for response to treatment. A study by Corpechot et al. on 150 patients showed that an increase of 2.1 kPa/year in LSM values is associated with an eightfold increase in the risk of developing further disease complications [32].

**Figure 1:**
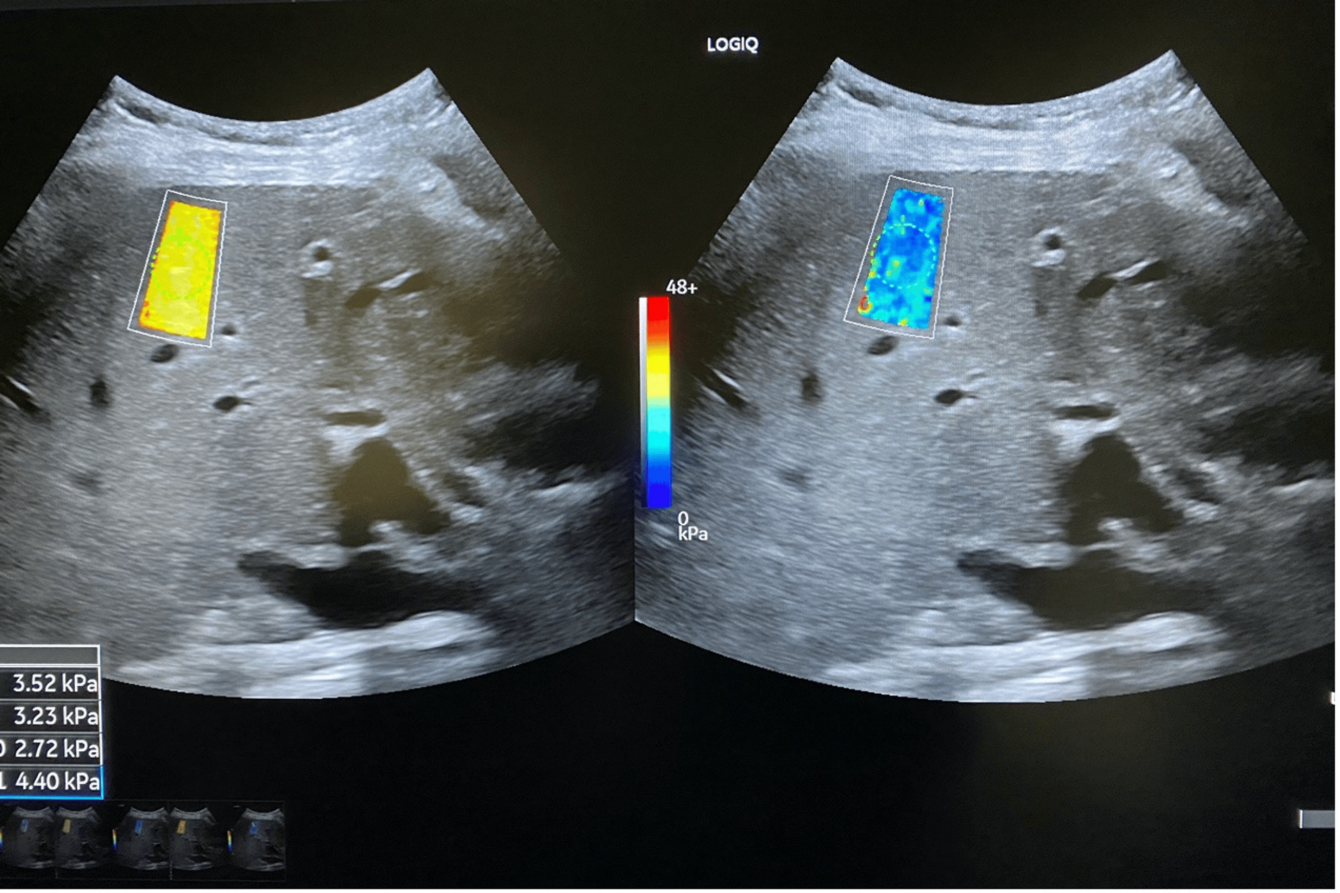
Normal 2D-shear wave elastography The ultrasound images shown are part of our team's original work and were obtained by the authors as part of this study.

Historically, in clinical examination, palpation has been a fundamental diagnostic tool for detecting various pathologies. The identification of stiff or hard masses often indicates underlying disease processes. This principle remains relevant in modern medicine, particularly in the context of liver fibrosis, where disease progression is characterised by gradual changes in tissue stiffness [25]. The ability to non-invasively measure tissue stiffness presents a valuable tool for diagnosis, staging, and management of hepatic conditions. Conventional ultrasonography has long been a widely accessible imaging technique for screening and diagnosing liver diseases, owing to its non-invasive nature, cost-effectiveness, and absence of ionising radiation. However, its limitations in subjectivity and moderate accuracy, especially in cases of mild steatosis, have prompted the development of more advanced techniques. The ultrasound-guided attenuation parameter (UGAP) addresses these shortcomings by combining ultrasound image guidance with elastography (2D-SWE) and attenuation coefficient (AC) measurement [34]. Two-dimensional shear wave elastography (2D-SWE) represents a significant advancement in non-invasive liver stiffness assessment. This technique complements traditional ultrasound imaging by quantifying tissue elasticity and hardness, offering an additional perspective on liver parenchymal health. 2D-SWE is simple, painless, and provides real-time measurements, making it an attractive option for clinical practice [35]. Shear wave elastography (SWE) has emerged as the most reliable among various elastography methods. It utilises shear waves to assess and quantify tissue elasticity. The propagation of these waves, approximately 10,000 times faster than conventional ultrasound waves, allows for rapid data acquisition. The elasticity measurements are visualised as a colour-coded overlay on the standard 2D ultrasound image, with measurements typically expressed in kilopascals (kPa). In these coded images, harder tissues are represented in red, while softer tissues appear in blue. Despite some technical limitations related to shear wave propagation, 2D-SWE currently stands as the only elastography technique capable of providing reliable quantitative and qualitative information about examined tissues in real time. This capability makes it an invaluable tool in the assessment of liver fibrosis, complementing other non-invasive methods and potentially reducing the need for invasive liver biopsies in certain clinical scenarios [25]. Normal, non-fibrotic, non-steatotic liver tissue as examined under 2D-SWE can be seen in Figure 1.

How elastography is performed

The shear wave generated by the transducer provides real-time imaging of lateral tissue deformation, allowing for immediate assessment of wave propagation. Using a conventional transducer, pressure waves are produced, and a series of images are captured to create a programmed beam. After measuring the local propagation speed, a two-dimensional map is generated, offering a quantitative estimate of tissue stiffness represented in kilopascals (kPa). 

While shear wave elastography (SWE) is a non-invasive procedure, it has certain limitations. For instance, stiffness measurements of the pectoralis major muscle are not necessarily indicative of fibrosis, as various factors can influence elastography results. Conditions such as acute hepatitis may yield false-positive results, and in obese patients with high body mass index (BMI), inaccurate values may be obtained. Additionally, factors such as oedema, inflammation, cholestasis, and congestion can confound results [23]. 

The framework shown in Table 1 allows clinicians to interpret liver stiffness measurements effectively and make informed decisions regarding patient management. 

In summary, while SWE offers a promising non-invasive approach to assess liver stiffness and diagnose conditions like fibrosis and cirrhosis, it is essential to consider potential confounding factors that may affect the accuracy of results. By integrating this technology into clinical practice, healthcare providers can enhance their diagnostic capabilities and improve patient outcomes in managing liver diseases [25].

Ultrasound-guided attenuation parameter (UGAP)

Since metabolic dysfunction-associated fatty liver disease (MASLD), according to numerous studies, has been shown to affect at least a quarter of the population with an increasing trend, the assessment of hepatic steatosis, fibrosis, and the severity of inflammation is essential to accurately assess the prognosis of liver disease. A new liver steatosis quantification system is the Ultrasound-Guided Attenuation Parameter (UGAP) using the Controlled Attenuation Parameter (CAP) as a reference method. CAP is another relatively new technique applied along with FibroScan, which allows the quantification of steatosis by measuring the attenuation of the ultrasound beam throughout the liver and has shown a good correlation with liver biopsy in adults [36].

As seen in Table 2, CAP allows for the evaluation of hepatic steatosis through the calculation of the CAP Score. The range of CAP Score values is measured in decibels per meter and lies between 100 dB/m and 400 dB/m, with variations according to the patient’s age [36].

**Table 2:** Corresponding CAP scores and degrees of liver steatosis

CAP Score	Degree of Steatosis	Percentage of Liver Tissue occupied by Fat
238-260 dB/m	S1	Less than 1/3 (11% to 33%)
260-290 dB/m	S2	Between 1/3 to 2/3 (34% to 66%)
290-400 dB/m	S3	More than 2/3 (>67%)

The ultrasound-guided attenuation parameter (UGAP) method quantifies the attenuation coefficient based on a reference model that mimics the known attenuation characteristics of liver tissue. The reference model (phantom) includes glass bead particles of attenuating materials with a known attenuation coefficient. In UGAP mode, the transmission and reception conditions are set to the same values used in the reference model, and the acquired liver echo profiles are offset against the reference data. As a result, compensated audio profiles only represent the wear and tear caused by attenuation. If the audio compensation profile is flat, the attenuation is the same as the reference model. UGAP includes an automated measurement algorithm to find and analyse the optimal measurement range [37].

**Figure 2:**
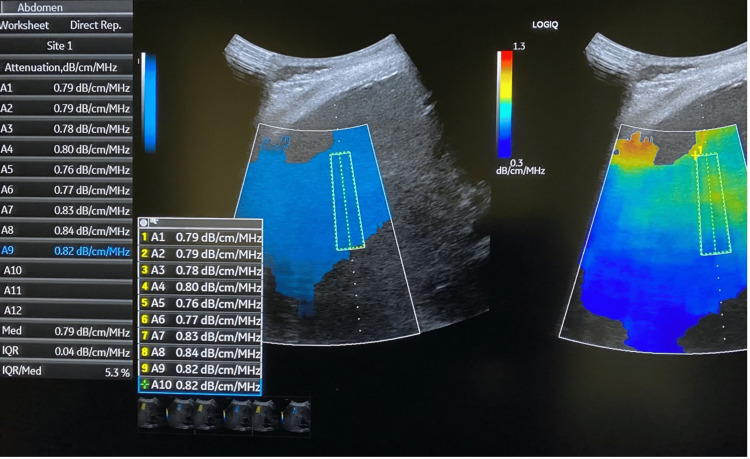
Ultrasound-guided attenuation parameter (UGAP) The ultrasound images shown are part of our team's original work and were obtained by the authors as part of this study.

To ensure objective measurements, UGAP technology employs a fixed depth of 4 cm, a frequency of 3.5 MHz, and a reference phantom model. By standardising the transmission and reception conditions of the ultrasound beam to match those used in the reference model, UGAP effectively assesses hepatic steatosis in patients with metabolic liver dysfunction. It analyses the differences between these signals to estimate beam attenuation within the liver parenchyma. For optimal examination, a region of interest is defined along the anterior axillary line in the right hypochondrium, strategically avoiding large blood vessels. The examination algorithm automatically excludes small structures, such as cross-sections of minor vessels, to enhance accuracy. In summary, UGAP serves as a valuable non-invasive tool for quantifying hepatic steatosis, contributing significantly to the assessment and management of metabolic liver disorders. Its reliance on standardised measurements and exclusion of confounding factors enhances its diagnostic utility in clinical practice. The UGAP method represents an important advancement in non-invasive imaging techniques for assessing liver health. By providing quantitative estimates of liver fat content, it aids clinicians in diagnosing and managing conditions such as metabolic dysfunction-associated fatty liver disease (MASLD). As non-invasive methods continue to evolve, UGAP stands out as a practical solution for evaluating hepatic steatosis, ultimately improving patient care through enhanced diagnostic accuracy [38]. An example of measuring liver tissue visualised using UGAP can be seen in Figure 2.

The application of 2D-shear wave elastography (2D-SWE) in liver assessment offers significant advantages, providing objective and reliable results. This technique is capable of detecting small lesions within the liver parenchyma that may be overlooked by conventional ultrasound. By accurately measuring and localising these lesions, 2D-SWE facilitates targeted biopsies when necessary. Furthermore, the straightforward implementation of this method enhances its diagnostic value in clinical practice.

When combined with the ultrasound-guided attenuation parameter (UGAP), 2D-SWE further improves the quantification of metabolic liver dysfunction, particularly metabolic dysfunction-associated steatotic liver disease (MASLD), and can guide clinical decision-making regarding specialist referrals and the necessity for liver biopsy.

**Figure 3:**
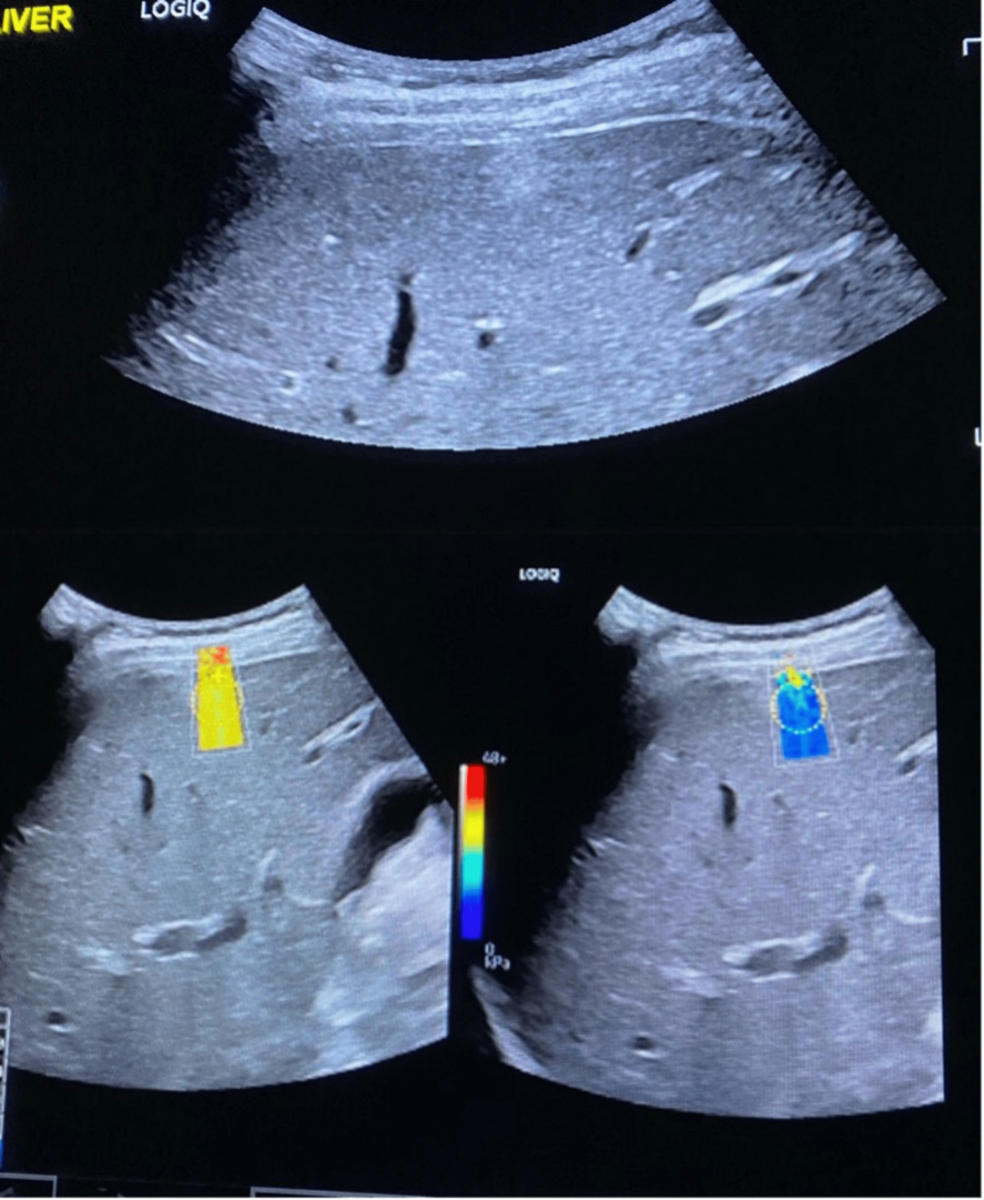
2D-SWE in non-malignant lesion (liver haemangioma) in the liver parenchyma (lesion stiffness 3.47 kPa) The ultrasound images shown are part of our team's original work and were obtained by the authors as part of this study.

**Figure 4:**
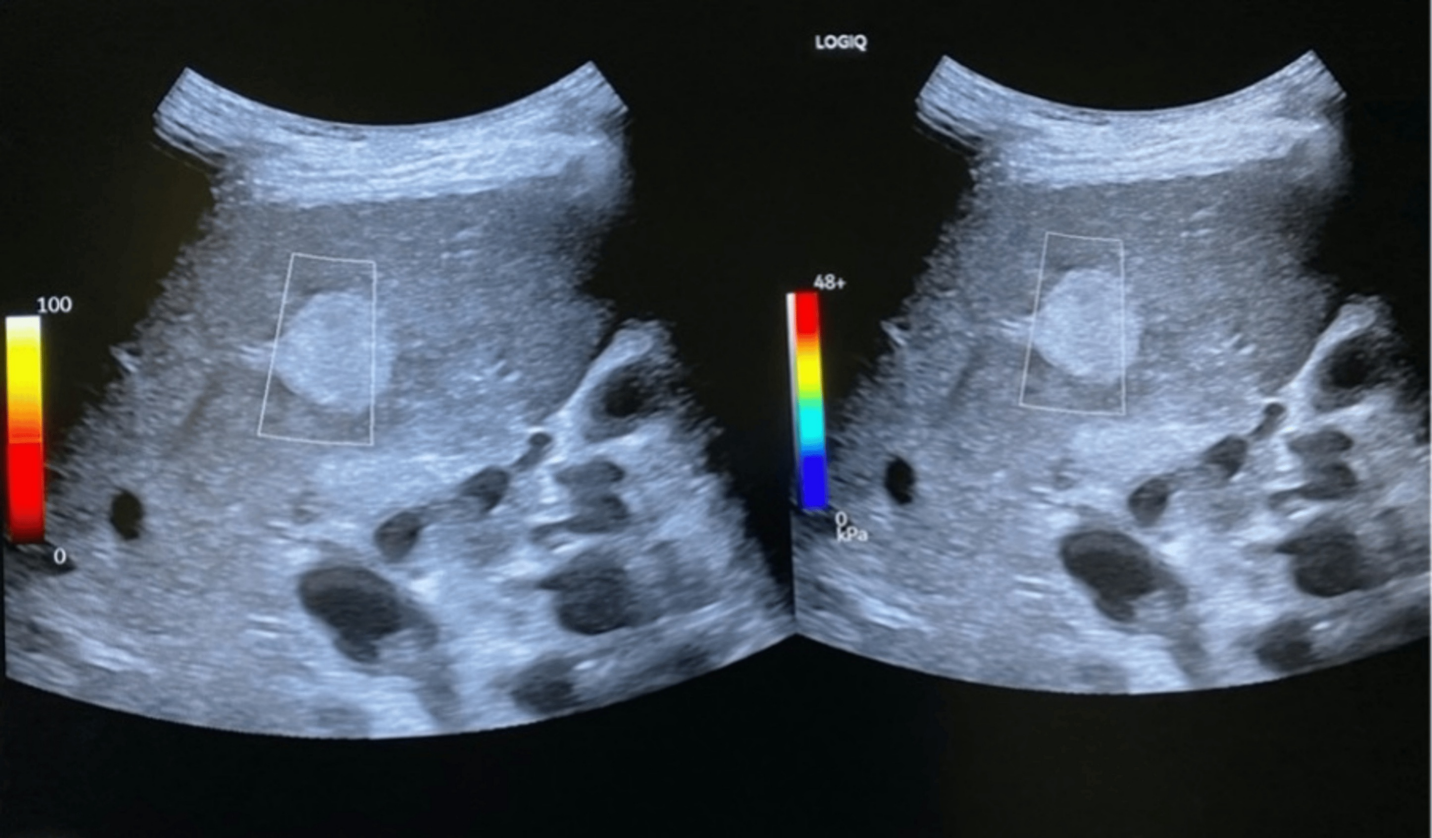
2D-SWE in malignant lesion in the liver parenchyma (lesion stiffness 24 kPa) The ultrasound images shown are part of our team's original work and were obtained by the authors as part of this study.

Recent studies comparing patients diagnosed with previously termed Non-Alcoholic Fatty Liver Disease (NAFLD), now classified as MASLD, have demonstrated that both 2D-SWE and vibration-controlled transient elastography (VCTE) yield equivalent results in assessing liver fibrosis. However, UGAP has shown superior performance in detecting steatosis grades ≥S1, ≥S2, and S3, with areas under the curve of 0.89, 0.91, and 0.92, respectively, significantly outperforming controlled attenuation parameter (CAP) measurements (P < 0.05). Thus, the combination of 2D-SWE and UGAP proves beneficial for stratifying risk in patients with progressive non-alcoholic steatohepatitis and aids in selecting candidates for specialist evaluation [39]. Examples of non-malignant and malignant lesions found in the liver parenchyma under 2D-SWE examination can be seen in Figures 3, 4.

In summary, the integration of 2D-SWE and UGAP into clinical practice enhances non-invasive evaluation methods for liver health. These techniques provide valuable insights into hepatic conditions, improving diagnostic accuracy and patient outcomes in managing liver diseases [40].

## Conclusions

Two-dimensional shear wave elastography (2D-SWE) and ultrasound-guided attenuation parameter (UGAP) represent a significant leap in non-invasive liver assessment in clinical practice. Through these methods, it is possible to detect and characterise metabolic dysfunction-associated fatty liver disease (MASLD). Additionally, they contribute to the early detection of metabolic dysfunction-associated steatohepatitis (MASH). Early diagnosis enables timely implementation of lifestyle modifications, including dietary changes and increased physical activity, which can potentially halt disease progression and potentially reverse liver damage in the initial stages of MASH. 2D-SWE also demonstrates utility in the differential diagnosis of malignant liver tumours, aiding in the characterisation of focal lesions within the hepatic parenchyma. The combination of 2D-SWE and UGAP provides a comprehensive assessment, allowing for risk stratification of progressive metabolic dysfunction-associated steatohepatitis (MASH). This integrated approach can guide clinical decision-making regarding the necessity for specialist referral and liver biopsy, optimising patient management and reducing the need for invasive procedures in select cases.

As these technologies continue to evolve, their widespread adoption has the potential to significantly impact the early detection, monitoring, and management of various liver diseases, ultimately improving patient outcomes and reducing the burden of advanced liver disease. The ability to non-invasively assess the liver as an organ represents a paradigm shift in hepatology, paving the way for more personalised and effective interventions.
